# Being a legal guardian – the nursing perspective

**DOI:** 10.1186/s13584-015-0056-1

**Published:** 2015-11-24

**Authors:** Michael Kuniavsky, Ilana Kadmon, David Chinitz

**Affiliations:** General ICU, Assaf Harofeh Medical Center, Beer Jaacov, 70300 Israel; The Hebrew University School of Public Health and Community Medicine, Faculty of Medicine, Jerusalem, Israel; Hadassah-Hebrew University School of Nursing, Faculty of Medicine, Jerusalem, Israel

**Keywords:** Legal guardian, Nurses, Surrogate decision making, Qualitative research

## Abstract

**Background:**

Surrogate decision making is common in public healthcare worldwide. In Israel any incompetent adult patient requires a Legal Guardian (LG), appointed by the court, for approval of invasive none-life saving procedures. Usually, the LG is a close family member of the patient. Nurses are the most available healthcare providers to the families and the LG during the process of appointment and afterwards. The patient's family is often anxious or even depressed, and thus the perceptions and behavior of nurses charged with providing support are crucial.

In a previous study based on interviews of LGs we found that the most difficult issues for the LGs were decision related issues, family related issues and appointment bureaucracy issues.

**Objective:**

To qualitatively assess nurses attitudes regarding the difficulties that families and LGs face during and after appointments and to compare the findings to previously accessed LG attitudes.

**Research design:**

After IRB approval, demographic and semi-structured questionnaires were used to assess the attitudes of a convenience sample of 34 nurses who were participating in a critical care training course (41 % of the respondents were from the ICU, 47 % from medical or surgical wards, and 12 % from other departments at secondary and tertiary hospitals in Israel.) regarding LGs difficulties. After reading and analyzing the responses provided by the nurses, the authors categorized the pertinent topics raised using content analysis. Nurses' perceptions were also compared to those of LGs reported in previous research by the authors.

**Results:**

Three main themes emerged: 1. Decision related issues; namely coping with the complexity of end of life decision issues; 2. Family related issues; namely, family dynamics related to the various decisions regarding LG identity and patient care; and 3. Bureaucracy issues; namely, the formal process related to LG appointment and decisions. Regarding the first two themes, the feelings of the nurse respondents were quite similar to those of LG respondents from our earlier research. The third theme - bureaucracy issues – was never mentioned by the nurses, as opposed to LGs who mentioned it frequently. This suggests that the nurses did not consider it to be an important issue.

**Conclusions:**

The difficulties of decision making as well as family support and responsibility of LGs are well known by nurses. The appointment and bureaucracy issues were neglected by nurses, although they are very important to the LGs. Improvement of this parameter of care is needed. Possible directions for improvement include raising awareness of nurses regarding the appointment process and alleviation of bureaucracy. Further research is required to identify appropriate strategies for improving these aspects of care.

## Introduction

In worldwide medical practice informed consent is a prerequisite for almost every invasive procedure, and is a crucial part of patient rights. Often the patient is unable to give such informed consent for a variety of reasons (mentally incapable, minor, unconscious, sedated, etc.). In such cases, in order to preserve patient autonomy, different countries have developed different approaches. Those approaches are articulated in laws, regulations or guidelines [1–5]. Some demand that the decision be made by the physicians (paternalist approach), some demand that family take over and decide for the patient (autonomous approach) and lately there has been developed a shared decision approach wherein a combined family-medical team decision is made [1, 3, 5, 6]. The issue, debated worldwide, is universal on one hand but unique to each country, on the other [7, 8].

In Israel, legal guardians (LGs) are appointed by law when a patient is unable to give informed consent, except for immediate life threatening emergency cases. In such cases three treating physicians sign informed consent papers on behalf of the patient. In all other cases the patient's family makes a recommendation to the courts about the LG’s identity, usually under guidance of a hospital social worker. The designated family member then applies to a court of law with recommendation from a hospital social worker and attending physician and needs to pay a court fee. When the patient is unconscious no formal specialist consultation (e.g.: psychiatrist, geriatrician) needed. The court decides regarding LG identity, usually approving the designated family member. The court of law also decides for how long and for what procedures to allow the LG to be the patient’s representative.

**Fig. 1:**
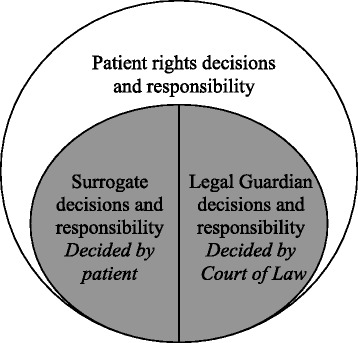
Model of patient, surrogate and LG decisions and responsibilities

The whole procedure of LG appointment takes at least a few days and might take up to weeks. Usually, the appointed LG is a patient's close relative. This legal procedure is based both on the Legal Capacity and Guardianship Law (1962) [9] and Patient Rights Law (1996) [10]. The process occurs on a daily basis in every Israeli hospital. Another possibility, much less widespread, based on the Law of the Dying Patient (2005) [11] and in part on Patient Rights Law (1996) [10] allows a competent patient to formulate advanced medical directives, [Law of the Dying Patient (2005) [11]] or appoint a family member to have power of attorney in order to make medical decisions on his or her behalf in the future [Law of the Dying Patient (2005) [11] and/or Patient Rights Law (1996) [10]]. This option is currently in very rare use, and demands certain bureaucratic procedures to be taken by the patient, while he or she is competent. These options are represented in Fig. 1.

Studies reveal that patients' families are under significant tension which results in anxiety, depression and even symptoms of post-traumatic stress (PTSD) [12–15]. Moreover LGs often do not know patients’ preferences regarding the procedures needed to be taken, making the decisions LGs face even more complex and difficult [1, 15]. It is the physician's responsibility to provide the information about the diagnosis, prognosis and the treatment risks and benefits to the patient and his or her relatives. Nevertheless bedside nurses are the staff members most available to the family and the LG during the process of LGs appointment. Therefore LGs, like all other family members of the patient, tend to rely on the nurses to provide and/or explain most of information given during this stressful period of time [15–19]. In addition, nurses are a valuable and accessible source of explanations for information handed over by the physicians for the patient, the family and the LG. The patient's family is often anxious or even depressed, and therefore in need of detailed and gradual guidance provided by nurses [12–17].

As a result, the nurses’ point of view regarding the process of LGs appointment and difficulties that LGs encounter during this process is important. Even if they are not dominant in the families' decision making processes , their proximity makes them a good source of information about the processes. Moreover, nurses inevitably exert some influence on family decision making, so knowledge of their perceptions can contribute to improving the process of LG appointment and functioning.

Despite the importance of the issue, research in the field is scant and knowledge of nursing attitudes is minimal, if any. The knowledge of nursing attitudes regarding LGs difficulties will allow better understanding of the process that has taken place in Israel on a routine daily basis for almost 20 years. Through this understanding, it is possible to identify areas where nurses' knowledge or attitudes may be improved. As a result, nurses will be able to improve assistance to LGs in their complex decision making process.

The current research objective is to check with the nurses what, in their opinion, are the most significant difficulties for LGs, during the decision making process. The second objective of the research is to compare these findings to LGs’ difficulties that were found in our previous studies [1, 2]. Those studies used both quantitative [1] and qualitative [2] approach and revealed three main themes LG found most difficult: decision making issues, family related issues and bureaucracy issues regarding appointment.

## Methods

A convenience sample of thirty four nurses from different working places and different demographics were enrolled for the study after receiving Institutional Review Board approval. The data was collected anonymously in writing during September and October 2012, from nurses who were in the midst of a Ministry of Health critical care nursing course. Of 46 participants in the course, 34 completed the questionnaire. Forty one percent of nurses who agreed to participate worked in the ICU, 47 % in medical or surgical wards and 12 % elsewhere. Participants came from a variety of medical institutions.

The study included both a demographic questionnaire (age, gender, education, marital status etc., see Table 1) and an open question: “Recall one of the LGs you have met in your practice and tell what difficulties he or she encountered?”. In case the respondent failed to describe LGs’ difficulties, a clarification question was used: “Imagine yourself in the position of the LG you described, and tell us what difficulties you think you would encounter?”. We made a concerted effort, as we did with the LGs in the previous study, not to guide the respondents too much, and wished to uncover whether nurses would raise the same issues as LGs without prompting. They were asked to complete the demographic questionnaire (see Table 1). In addition, they were asked to answer, in writing, within a few weeks, the above two questions:

**Table 1:** Respondents demographic data (N = 34)

Age	
Average in years (± SD)	34.42 (±6.35)^a^
Gender	
Female	71 %
Male	29 %
Marital status	
Single	35 %
Married	53 %
Divorced	12 %
Religion	
Jewish	76 %
Muslim	9 %
Christian	3 %
Other	12 %
Religiosity	
Religious	17 %
Conservative	17 %
Secular	62 %
Other	3 %
Education	
BA	79 %
MA+	21 %
Working place	
ICU	41 %
Ward	47 %
Other	12 %
Professional work experience	
Average in years (± SD)	5.38 (±3.67)

^a^One respondent omits age data (therefore for this parameter *N* = 33)

**Fig. 2:**
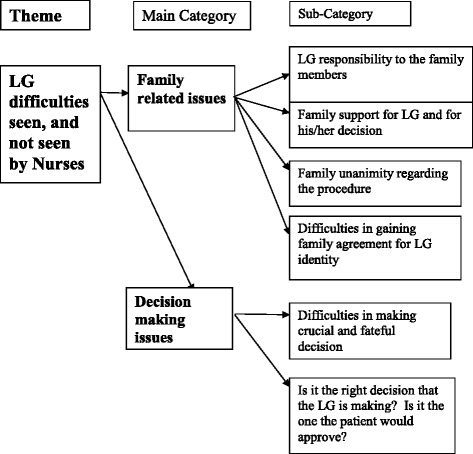
Content analysis scheme

After receiving the data, descriptive statistics were produced for the demographic data: frequencies, measures of central tendency and dispersion were analyzed using the SPSS 14 statistical package. The open questionnaire responses were categorized. The content analysis [20, 21] was performed by one of the authors (MK) by hand, after reading, re-reading and analyzing the answers provided by the respondents. Meaning units were identified in the text and the author (MK) arranged them in categories named to capture the pertinent topics that respondents raised in their answers [20, 21]. As often happens in qualitative research, categories spawned sub-categories that enriched the findings. The categories and subcategories were organized in a diagram (Fig. 2) that enabled consideration of the elements of the LG process and their interaction. These are described in the findings section.

## Results

The study was conducted during September and October 2012. A total of 34 nurses were asked and agreed to participate in the study. Convenience sampling was used. Most of the respondents were female nurses (71 %) with first (79 %) or second (21 %) degree university education, in their mid-thirties, married (53 %), Jewish (76 %) and secular (62 %), working in wards (47 %) or ICU (41 %) for more than 5 years as nurses. Demographic data of the respondents presented in Table 1.

**Table:** 

An example of the LG experience, as described by a respondent A 73 year old male patient, married with children, was admitted to the ICU after pre-hospital CPR. During a month-long hospitalization, the patient remained intubated and ventilated without ability of spontaneous breathing. After medical advice regarding performance of a tracheotomy was discussed with the children and spouse of the patient, and the need for LG appointment was clarified, the family felt very reluctant to take on the responsibility. The daughters of the patient responded in a highly emotional way to that suggestion. They declined the option to become the patient’s LG on the grounds that they lacked the physical and emotional strength for such a task. One of the sons felt very reluctant to undertake the role and make the decisions for his father. In the end, the whole family (sons, daughters, spouse) nominated one of the sons to be the LG, although he was very reluctant, and asked numerous questions such as: Why me? What this will demand from me etc.? After the whole family backed and approved him he agreed, filled out the necessary papers and 3 days later was appointed as Legal Guardian for his father by a court of law. A few days later he made the decision and the tracheotomy procedure was performed (respondent 7).	

Respondents described a variety of stories like the one in the above box, mostly about unconscious patients in need of a Legal Guardianship decision, and their families. By way of introduction to the findings it should be said that the data gathered from the nurses in the sample presents an irony. While the tumultuous context in which the families find themselves are richly described, the nurses make only limited reference to what they imagine might have transpired in the bureaucratic/legal process of appointing an LG. The respondents describe well and express empathy for the emotional stress of families and LGs, the gut wrenching decisions faced by them, and the complex interactions among family members that take place. This “boundedness” of nurses’ perceptions regarding the families, that extend only to the door of their department but do not follow the families outside comes through in the textual analysis, as described in the methods section, that follow. The descriptions by respondents of the processes surrounding Legal Guardianship; namely, the situation that creates the need for a LG, the selection process, and the behavior of LGs and their families, revealed the categories displayed in Fig. 2. The main categories uncovered were “Family Issues,” and “Decision Related Issues”.

### Category: Family Issues

- LG responsibility to family members

Nurses were keenly aware of the intra family dynamics that were at play in the richly described situations the families found themselves in. Respondents stressed the importance of LG responsibility to family members. The need to be the one that explains the consequences of the decision to the family was raised in respondents' answers. The rapid deterioration of the patient threw the families into crisis and lack of balance. The respondents described a shared perception among family members of the role of the LG, both during the process of selecting the latter, and once the responsibility had been assigned. Yet, evidently, the nurses did not connect the stress, uncertainty and felt weight of responsibility to the process of appointment of the LG.

*“LG difficulties were many, mainly confronting the fears of family regarding the deterioration of the patient condition and possible death. The relatives were under stress and LGs had the obligation to alleviate their uncertainty.” (15)*

- The need for family support for each other and for the LG

The nurses observed that LGs were in need of knowing that the families were behind them. Respondents described the importance of family support during the decision making process. Relatives' opinions and support were of great importance for LGs from the respondents’ point of view. The family was seen as being in a process of coming together to confront the situation and choosing someone to lead them.

“*It was only the coming together of the whole family that enabled the LG to answer the question (2)*

“*Will my decision be accepted by the family?*”(8)

“*This also was manifested in family’s decision, after the death of their mother, to undergo genetic testing for the disease that took her.”(2)*

- Family unanimity regarding the procedure

The general coming together of the family and support for the LG found expression in coalescence around the specific decisions that had to be made, and the role of the LG in tying family unity to those decisions. Respondents stressed the importance of family unanimity regarding performance of invasive procedures. The respondents’ opinion is that unanimity of the family members is important.

“*The LG faces the difficulty of uniting the family in order to reach a unanimous decision. “ (15).*

“*After considering options, rethinking and consultations, the family came to a unanimous decision regarding the treatments of the patient. The decision was a tough one, but since it was a unanimous decision by all close relatives of the patient the responsibility was shared among all of them” (4)*

*“In the end they reached a unanimous decision regarding the treatment and the LG appointment.”(2)*

“*The excellent relationship of all the family members together allowed them to reach a unanimous decision” (12)*

*“This only happened after the family members reached deep unity” (2)*

- Difficulties in gaining family agreement for LG identity

Respondents stated that the selection of the LG is a complex, difficult issue. It is not clear who is the suitable person for the task. Sometimes no one is willing to undertake the role.

*“After the need for LG appointment was clarified, the family felt very reluctant to take on the responsibility. The daughters of the patient responded in a highly emotional way to that suggestion. They declined the option to become the patient's LG on the grounds that they lacked the physical and emotional strength for such a task. One of the sons felt very reluctant to undertake the role and make the decisions for his father. In the end, the whole family (sons, daughters, spouse) nominated one of the sons to be the LG, although he was very reluctant.”(7)*

*“In the beginning the family was disputed regarding the treatment options and LG identity” (2)*

### Category: decision related issues

- Difficulties in making crucial and fateful decisions

Respondents were well aware of the complexity and difficulty of the decision LGs have to take. They state it is a scary and very difficult task for LGs.

*“Could my decision endanger my spouse?”(8)*

*“The LG is in fear of both the patient’s deteriorating condition and the prospect of death “(9)*

*“The LG fears the consequences of the decision*” (13)

*“The real difficulty of the LG is to make a decision than could be one of life or death, or at least influence the quality of the patient’s life” (10)*

*“The LG faces a dilemma: to agree for the treatment or not. In either case the LG will need to suffer the consequences of her decision if the patient’s condition deteriorates or if the patient improves but turns out to disagree with the decision LG made” (11)*

*“The difficulty is to be responsible to make a decisive and far reaching decision in favor of an invasive procedure that could damage the patient’s body” (1)*

*“It is very difficult for LGs to make the decision. LGs are taking the responsibility for patient destiny. Moreover, the responsibility of the LG is enormous: to take life and death decisions regarding the patient.” (9)*

- Is the LG making the right decision?

Besides having to make the difficult decision, LGs were seen by respondents as needing to consider patient preferences that were often unknown to them. This was stated clearly by the respondents in their answers.

“*Why me, am I the right person?”(8)*

*“The decisions that LG making are not always what the patient really wanted.”(9)*

What is remarkable is that despite the obvious close observations that respondents could make regarding family conditions and behavior, they did not refer to what transpired in the legal process of appointing the LG. It is the combination of the rich understanding nurses demonstrate regarding what their patients and clients have gone through, together with their obliviousness to the appointment process that gave us our major theme; namely, what nurses know and do know about the LG process. The implications are taken up in the Discussion section.

## Discussion

The surrogate decision making process is a challenge for the patient’s family, the LG and the medical team. It aims to preserve patient autonomy right through the surrogate decision maker involvement. Nurses are the team members most available to patients, families, and Legal Guardians, thus making their knowledge and attitudes important.

Our study shows nurses are well aware of family related issues such as family unanimity, family support and responsibility of the LG towards the family members. The importance of family related issues to surrogate decision making is well documented by numerous studies. Family members and close relatives play a crucial role for the LG. Their support makes it easier to make decisions and share responsibility. Family conflict makes the LG role more complicated [22–24]. Moreover this correlates with LGs’ opinions in our previous studies, where LGs specifically stated the importance of family support and unanimity [1, 2]. However, in contrast to the research reported here, LGs did not feel difficulties regarding family agreement regarding choice of LG. Rather, they felt support from the family without arguments regarding the LG identity [1, 2]. We may conclude LGs are much more concerned about the appointment bureaucracy than about family issues therefore making the disparity between nurses and LG point of view even more important. On the other hand these differences could be due to bias of the respondents in the current study. Nurse respondents usually related in their answers to the most difficult or colorful case they remembered. LGs, however, do not report frequent family disagreement regarding LG identity but, rather, unanimity [1, 2]. Further research is needed to fully investigate this issue.

Another theme, well known in surrogate decision research, that arises from our study, concerns issues related to decisions by LGs. The decisions surrogates need to make are difficult. This might cause severe burden, especially when the patient’s wishes are unknown. In cases where the surrogate is aware of the patient’s wishes, decision making is easier [12, 14, 22, 23, 25]. The nurses interviewed in this study, as well as the LGs in our previous study, were unanimous in their feelings that LGs face difficult and crucial decisions. Nurses also demonstrate awareness of the situation in which it is not clear for LGs what the patient would prefer or approve, making decisions even more complicated [1, 2, 14]. Regarding this issue, nurses and LGs expressed similar feelings. Therefore we may conclude nurses are well aware of these difficulties perceived by LGs. Advance directives could be a possible remedy to this situation, if available and legally allowed [7, 26, 27]. Encouraging communication with the patient, within the family, and between family and medical staff regarding patient wishes is another possible course of action [14, 20]. However, when the patient is unconscious or incompetent it is too late for this crucial part of this process. Therefore, the action should be taken before the patient deteriorates severely, for instance in primary care settings by community health care providers. Further research is needed to find approaches to ease this complicated issue.

The key issue of bureaucracy related to the appointment by a court of law was stressed by LGs in previous studies [1, 2]. An official LG appointment is not needed in every case of surrogate decision making and depends on each country’s legislation [7]. Despite being a key issue to the LGs, who raised it on their own without prompting, it was completely omitted by the nurse respondents in the current research. The reason for this divergence might be that the appointment process is not proximate to the nurses. Nurses do not participate actively in the appointment process despite being the most available team member to address LGs concerns, as well as to guide and explain procedures that the latter is going to experience. The appointment process is held in the hospital by a social worker and the attending physician, and the actual appointment itself takes place outside the hospital in a court of law. Therefore, nurses are usually unaware of the difficulties of the appointment process. This process, although taking place on a routine basis for almost 20 years, may often be obscured to the nurses for the simple reason that it takes place distant from them and they are not involved in it.

This constitutes an important gap between the LGs feelings and attitudes and nurses’ knowledge. This could lead to improper nursing guidance and explanation to the family of the patient and to the LGs. If nurses are unaware of difficulties that lay ahead of the family members during the process of appointment by a court of law, they are unable to prepare the family and the future LG properly. This might cause excessive stress and anxiety reaction, even more than the family is dealing with already [13–16]. It is important and crucial to raise the awareness of the nurses to the issue.

The lack of nursing knowledge regarding appointment process could be improved if nurses would be more involved in LG appointment teams together with social workers and attending physicians. An alternative would be to have training workshops for active nurses on the entire process of LG appointment, provided costs in terms of time and money are not unreasonable. For nursing students, training regarding LG appointment should be integrated into nursing school curricula or qualification courses, thus making it less costly and available to wider range of nurses. Based on such training, nurses would take an active part in the process and would be aware of the LGs’ and families’ difficulties during the appointment process. Another remedy is to revise the whole process of LG appointment. If appointment could be waived in certain cases this would be a significant remedy to the families. However this issue, at least in Israel, demands legislative action.

Further research regarding best strategies for improving nurse awareness and nursing involvement in LG appointment process is advised.

### Study limitations

This research is the first to check nurses' attitudes towards LG difficulties. Data was drawn from a limited convenience sample of nurses participating in a special intensive care course. Regarding this sampling method, there are two issues to consider. First, while the sample included nurses from a number of Israeli hospitals and there is no a priori reason to think that the answers received would differ from those of a larger, systematically representative sample, this possibility cannot be ruled out. Further research, both qualitative and quantitative should be based on more purposeful and representative samples respectively. Second, we might assume that nurses exposed to such training would likely be more aware of LG issues than their peers who have not participated in such a course, but confirmation of this assumption would require further research.

Data gathering was based on open questions that were intended to be identical. It is marginally possible that the use of the phrase “decision processes for the LG,” was understood differently by the two groups of respondents from a linguistic point of view. Further research should include in-depth interviews, group or personal, to inquire regarding these aspects of respondents’ answers.

Respondents were free to choose any case of legal guardianship they encountered in their practice, without any limitation (e.g.: by ward, time, complexity of the case etc.) thus making a wide range of LG situations to be present in answers of the respondents. Most respondents described cases of unconscious patients, thus not representative of the whole population of patients evaluated for guardianship in general hospitals. In summary, having these initial findings regarding the nurses’ apparent lack of awareness regarding the LG appointment process, research should be carried out to understand the mechanisms obscuring the appointment process in the cognitive frame of the nurses.

### Challenges arising from the research

A LG is a patient representative appointed by a court of Law who is responsible for preserving the patient autonomy. In order to fulfill his or her stressful and difficult task, which is often unclear as the patient’s wishes are obscure, the LG needs assistance and guidance from the medical team. Nurses, as an important part of the team and the ones most available to the LG, are expected to give guidance and support during the process in order to meet the ethical standard of patient autonomy. We found that nurses are aware of LGs’ difficulties in decision making and family related issues. However our study also reveals a gap between LGs difficulties as LGs describe it as opposed to how nurses perceive it regarding bureaucracy issues during the appointment process. This gap may lead to improper guidance and might interfere with the fulfillment of the right to autonomy of the patient. Therefore, the issue must be taken into consideration in cases of official LG appointment processes worldwide.

### Suggestion for future research

We suggest research using group or personal in-depth interview with nurses regarding their point of view of LG difficulties as nurses see it. Another direction could be building a number of cases concerning legal guardianship (based on clinical cases or on in-depth interviews with LGs) that could be presented to nurses for learning and simulation. The respondents would be asked to describe what difficulties, in their opinion, LG encounter in situation described. Alternative types of action could be elicited from both LGs’ and nurses regarding what, in their opinion, are possible interventions to improve current situation.
